# GATA2 downregulation contributes to pro-inflammatory phenotype and defective phagocytosis of pulmonary macrophages in chronic obstructive pulmonary disease

**DOI:** 10.18632/aging.206129

**Published:** 2024-10-07

**Authors:** Shaoran Shen, Qiqing Huang, Lele Liu, Xiaoli Zou, Tutu Kang, Jianqing Wu

**Affiliations:** 1 Department of Geriatrics, Key Laboratory of Geriatrics of Jiangsu Province, The First Affiliated Hospital of Nanjing Medical University, Nanjing, Jiangsu 210029, China

**Keywords:** COPD, macrophages, phagocytosis, inflammation, Gata2

## Abstract

Pulmonary macrophages from COPD patients are characterized by lower phagocytic and bactericidal activity whereas there is hypersecretion of pro-inflammatory cytokines. The prominent decline of GATA2 expression in pulmonary macrophages from COPD patients inspired us to figure out its role during COPD development. The expression levels of GATA2 were decreased in alveolar macrophages isolated from cigarette smoke (CS)-induced COPD mice and cigarette smoke extract (CSE)-treated macrophages. *In vitro*, both CSE and GATA2 knockdown via siRNAs elevated pro-inflammatory cytokines expression whereas inhibiting phagocytosis in macrophages. Integrated analysis of transcriptomics of GATA2-knockdown macrophages and the results of ChIP sequencing of GATA2 together with dual-luciferase reporter assay identified *Abca1* and *Pacsin1* as functional target genes of GATA2. Mechanistically, ABCA1 mediates the pro-inflammatory secretion phenotype and the dysfunction in early stage of phagocytosis of macrophages through TLR4/MyD88 and MEGF10/GULP1 pathways, respectively. PACSIN1/SUNJ1 partially mediates the disruption effects of GATA2 downregulation on maturation of phagolysosomes in macrophages. Together, our study suggests that GATA2 influences multiple functions of pulmonary macrophages by simultaneous transcriptional regulation of several target genes, contributing to the dysfunctions of pulmonary macrophages in response to CS, which provides an impetus for further investigations of GATA2 or other underappreciated transcription factors as regulatory hubs in COPD pathogenesis.

## INTRODUCTION

As the most abundant innate immune cells in the lung, pulmonary macrophages significantly increased in the lungs of individuals with COPD though, they exhibit lower phagocytic and bactericidal activity whereas there is hypersecretion of pro-inflammatory cytokines [1]. Such pulmonary macrophages have been widely accepted as orchestrators of lung tissue inflammation during COPD development [2] by recruiting pro-inflammatory innate immune cells while activating adaptive immune cells, which could be aggravated due to decreased clearance of cellular debris and inhaled pathogens within lung tissues on the other hand [3]. Although many molecules and pathways necessary for dysfunctions of pulmonary macrophages have been identified in COPD pathogenesis [1, 4–6], vital transcriptional hubs for multiple dysfunctional responses of pulmonary macrophages remain less known.

Although GATA2 deficiency has long been regarded as a genetic disorder of hematopoiesis, lymphatics, and immunity though [7], more than half of these patients were reported to have pulmonary diseases, including pulmonary alveolar proteinosis (PAP), pulmonary hypertension, pneumonia, emphysema and bronchitis [8]. Impaired surfactant metabolism due to phagocytically dysfunctional alveolar macrophages (AMs) with unclear mechanisms underlies PAP, which indicates the crucial role of GATA2, a zinc finger transcription factor, in regulating pulmonary macrophage function besides in modulating endothelial and hematopoietic cells differentiation [9, 10].

Recently, with the advent of high throughput, next generation, and single-cell sequencing, GATA2 was found to be downregulated in pulmonary macrophages in several separate datasets from both COPD patients and smokers [11–13]. However, the exact influence of decline of GATA2 expression to pulmonary macrophages in COPD pathogenesis has been unexplored. This study validates the downregulation of GATA2 in both macrophages from CS-exposed COPD mouse model and multiple CSE-treated macrophage cell lines and further provides new mechanistic insight into the integrated role of GATA2 in regulating pro-inflammatory phenotype as well as phagocytosis of pulmonary macrophages.

## RESULTS

### GATA2 is downregulated in pulmonary macrophages during the development of CS-triggered COPD

**Figure 1:**
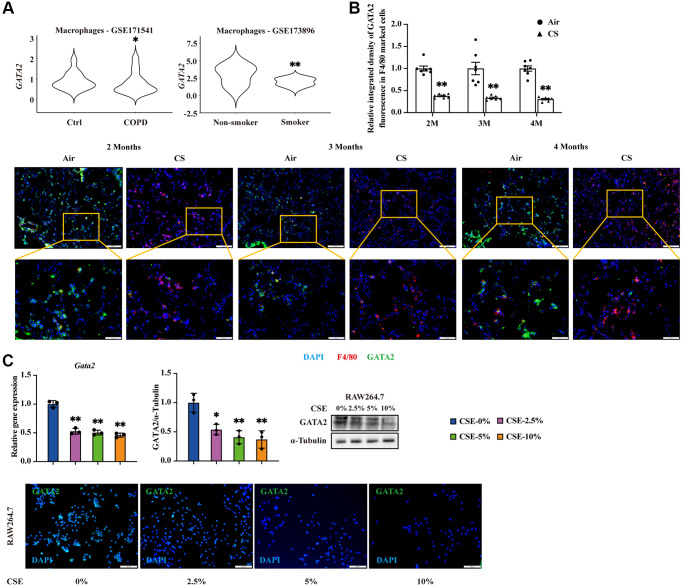
**GATA2 is downregulated in pulmonary macrophages during the development of CS-triggered COPD.** mRNA levels of *GATA2* were analyzed in database GSE171541 and CSE173896 (**A**). Eight-week-old mice were exposed to cigarette smoke (CS) or room air (*n* = 5–10). The lungs were harvested 2, 3, and 4 months after CS exposure and subjected to immunofluorescence staining of F4/80 and GATA2 (**B**). Scale bars = 50/100 μm. Relative integrated density of GATA2 fluorescence in F4/80 marked cells was measured from each group (Air, round dots; CS, triangle dots). mRNA and protein levels of GATA2 were measured by qPCR, western blotting, and immunofluorescence staining (**C**) in RAW264.7 cells treated with CSE for 24 h from each group (CSE-0%, blue bars; CSE-2.5%, magenta bars; CSE-5%, green bars; CSE-10%, orange bars). ^*^P < 0.05 or ^**^P < 0.01 vs. Air 5–10 animals per group or CSE-0%.

We first enquired the expression level of *GATA2* in macrophages from the lung tissues of COPD patients and smokers based on our previous single-cell RNA sequencing (scRNA-seq) dataset of human lung tissues from COPD patients and control donors [12] as well as that from smokers and non-smokers reported by Watanabe et al. [11]. *GATA2* was significantly downregulated in pulmonary macrophages from COPD patients and smokers (Figure 1A) compared to their matched controls, which were not observed in other cell types, such as endothelial cells, epithelial cells, NK cells, and T cells (Supplementary Figure 1A). To confirm the decline of GATA2 in macrophages during COPD pathogenesis, we established a COPD mouse model by CS exposure (Supplementary Figure 1B). Immunostaining of lung tissues from these mice showed an overlap of GATA2 and F4/80 (marker of macrophages) (Figure 1B). While F4/80 expression was increased in lung tissues of CS-exposed mice, reflecting the augmented macrophage infiltration and pulmonary inflammation, GATA2 expression was significantly decreased in both cytoplasm and nuclei of pulmonary macrophages compared to the control mice. Such decline in the mRNA level of *Gata2* was also observed in the primary AMs from bronchoalveolar lavage fluid (BALF) of CS-exposed mice (Supplementary Figure 1C). *In vitro*, we treated mouse monocyte macrophage leukemia cell line RAW264.7 cells and mouse alveolar macrophage cell line MH-S cells with different concentrations of CSE which showed no significant effect on cell viability (Supplementary Figure 1D) and found that GATA2 expression was downregulated at both mRNA and protein levels in a CSE dose-dependent manner (Figure 1C and Supplementary Figure 1E). Together, GATA2 is downregulated in pulmonary macrophages during the development of COPD.

### Downregulation of GATA2 leads to pro-inflammatory phenotype and impaired phagocytosis in macrophage

**Figure 2:**
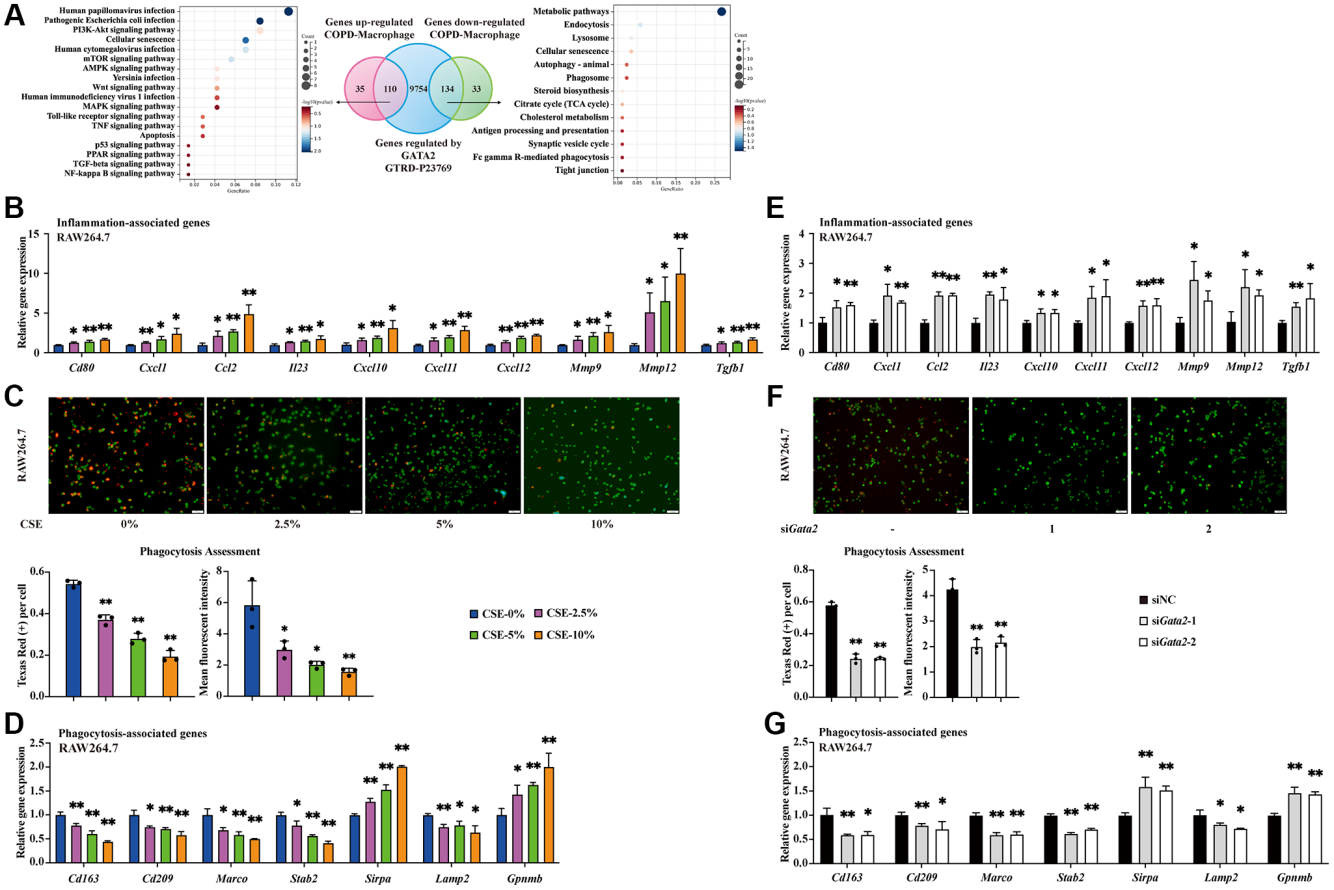
**Downregulation of GATA2 leads to pro-inflammatory phenotype and impaired phagocytosis in macrophage.** Differentially expressed genes from lung macrophages in COPD patients were intersected with GTRD database GATA2 ChIP-seq measurements and the genes were subjected to KEGG pathway analysis (**A**). mRNA levels of *Cd80, Cxcl1, Ccl2, Il23, Cxcl10, Cxcl11, Cxcl12, Mmp9, Mmp12,* and* Tgfb1* were measured by qPCR (**B**) in CSE-treated RAW264.7 cells from each group. Phagocytosis assessment in RAW 264.7 cells was done by Cell Meter^™^ Fluorimetric Phagocytosis Assay Kit. The images were taken using fluorescence microscopy and the average number of engulfed beads within every cell and the mean fluorescent intensity of Texas Red were calculated (**C**). Scale bars = 50 μm. mRNA levels of *Cd163, Cd209, Marco, Stab2, Sirpa, Lamp2,* and *Gpnmb* were measured by qPCR (**D**) in CSE-treated RAW264.7 cells from each group (CSE-0%, blue bars; CSE-2.5%, magenta bars; CSE-5%, green bars; CSE-10%, orange bars). ^*^P < 0.05 or ^**^P < 0.01 vs. CSE-0%. mRNA levels of *Cd80, Cxcl1, Ccl2, Il23, Cxcl10, Cxcl11, Cxcl12, Mmp9, Mmp12* and* Tgfb1* were measured by qPCR (**E**) in RAW264.7 cells from each group. Examination of phagocytosis was done by Cell Meter^™^ Fluorimetric Phagocytosis Assay Kit (**F**). Scale bars = 50 μm. mRNA levels of *Cd163, Cd209, Marco, Stab2, Sirpa, Lamp2,* and *Gpnmb* were measured by qPCR (**G**) in RAW264.7 cells from each group (siNC, black bars; si*Gata2*-1, gray bars; si*Gata2*-2, white bars). ^*^P < 0.05 or ^**^P < 0.01 vs. siNC.

Next, to investigate the influences of GATA2 dysregulation on pulmonary macrophages in COPD pathogenesis, we intersected the potential targets of GATA2 identified by ChIP sequencing (ChIP-seq) from GTRD database [14] with COPD-associated differentially expressed genes (DEGs) from pulmonary macrophages in our previous COPD human lung tissue scRNA-seq dataset. KEGG pathway analysis showed that the intersected genes upregulated in pulmonary macrophages from COPD patients were enriched in infectious and inflammatory pathways (PI3K-Akt, mTOR, Toll-like receptor, etc.), and that those downregulated ones were enriched in endocytosis, phagocytosis, lysosome, and phagosome (Figure 2A). Accordingly, we hypothesized that GATA2 downregulation may disrupt the macrophage phagocytosis and aggravate pulmonary inflammation by enhancing the expression of pro-inflammatory cytokines in macrophages during COPD development. Notably, inflammation-related molecules (*Cd80, Cxcl1, Ccl2, Il23, Cxcl10, Cxcl11, Cxcl12, Mmp9, Mmp12,* and* Tgfb1*) exhibited a dose-dependent increase in CSE-treated RAW264.7 cells (Figure 2B) and MH-S cells (Supplementary Figure 2A), concurrent with the decrease of GATA2 *in vitro* (Figure 1C and Supplementary Figure 1E). Meanwhile, we also examined the phagocytic function of CSE-treated macrophages using Protonex^™^ 600 Red Latex Beads. Generally, the average number of engulfed beads within every cell indicates the engulfing ability of macrophages, while the fluorescence intensity of phagocytosed beads, that increases as pH decreases from neutral to acidic during phagolysosome formation and maturation from phagosomes, reflects the digestion extent of the ingested particles in macrophages. As shown in Figure 2C and Supplementary Figure 2B, both the number of beads swallowed by RAW264.7 cells and MH-S cells as well as the fluorescence of beads within these cells decreased with the increase of CSE concentrations, suggesting that CSE treatment may injure the processes of ingestion and phagolysosome formation of macrophage phagocytosis. At mRNA levels, alterations of molecules vital to phagocytosis (*Cd163, Cd209, Marco, Stab2*, and *Sirpa*) and genes related to lysosome injury (*Lamp2* and *Gpnmb* [15]) all revealed phagocytic dysfunction of macrophages in a CSE dose-dependent manner (Figure 2D and Supplementary Figure 2C). Subsequently, we knocked down *Gata2* in RAW264.7 cells using small interfering RNAs (siRNAs) (Supplementary Figure 2D) and found that *Gata2* knockdown resulted in impaired phagocytosis of RAW264.7 cells as reflected by decreases of both the number and fluorescence intensity of engulfed beads (Figure 2F). Additionally, the increased expression of inflammation-related molecules and the dysregulation of genes involved in phagocytosis were also detected in *Gata2*-silenced RAW264.7 cells (Figure 2E, 2G). Notably, silencing *Gata2* in RAW264.7 cells alone was sufficient to partially recapitulate the effect of CSE on inflammation and phagocytosis in macrophages (Figure 2B–2D). Thus, downregulation of GATA2 led to pro-inflammatory phenotype and impaired phagocytosis in macrophage, especially the processes of ingestion and phagolysosome formation.

### Identification of *Abca1* and *Pacsin1* as transcriptional targets of GATA2 with inflammatory and phagocytic regulatory potential

**Figure 3:**
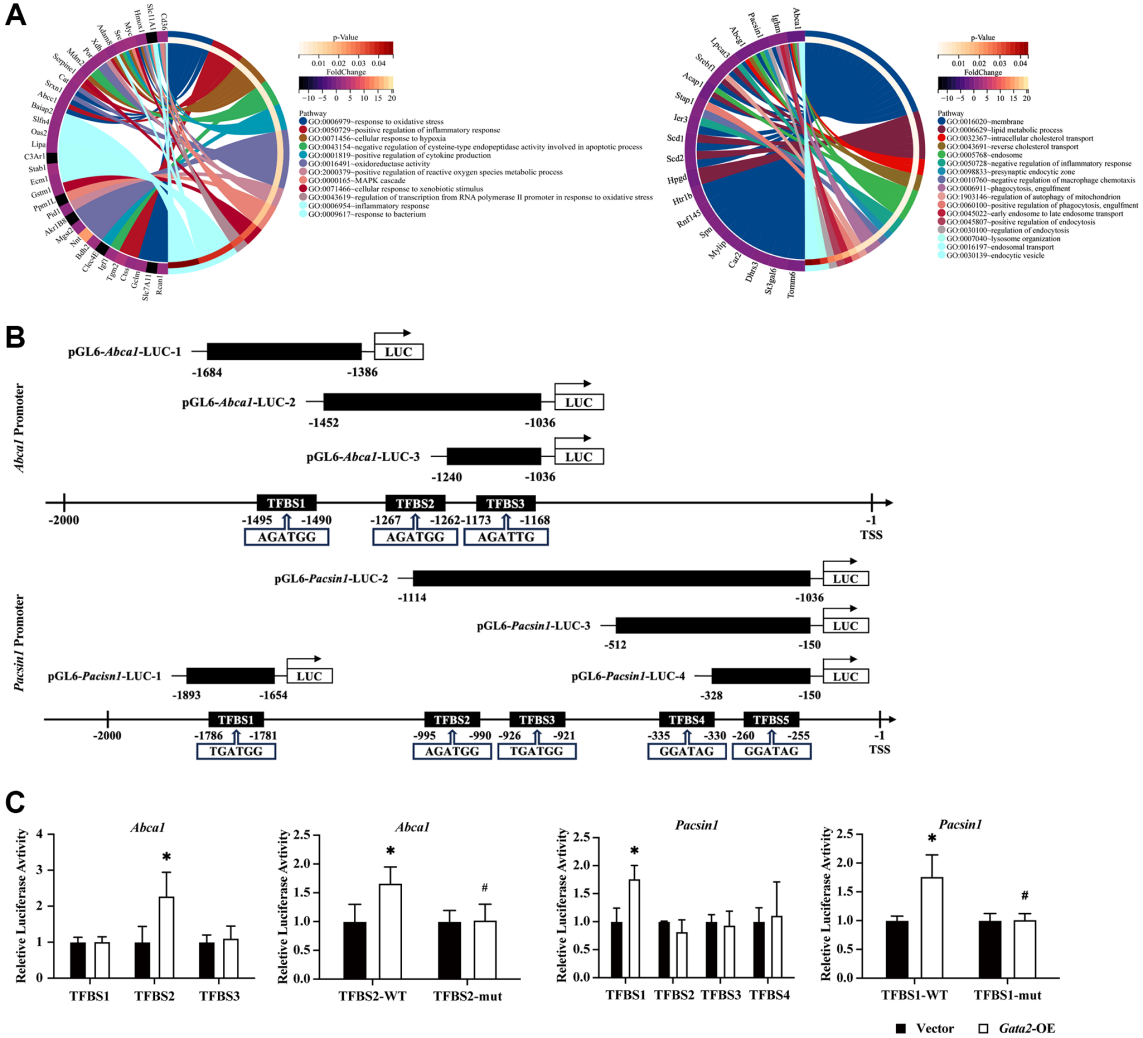
**GATA2 directly promotes the transcriptional activity of *Abca1* and *Pacsin1*.** GO enrichment analysis was done for upregulated/downregulated differentially expressed genes and different GO pathways and the related DEGs were connected with different colored bars (**A**). Schematic drawing of the mouse *Abca1/Pacsin1* promoter region with potential GATA2 DNA binding sites (black boxes) and that of the pGL6-*Abca1/Pacsin1*-Luc construct containing the *Abca1/Pacsin1* promoter region (**B**). They were co-transfected in mouse aortic endothelial cells (MAECs) and relative luciferase activity was measured. The mutant plasmid was then constructed and co-transfected, the relative luciferase activity was measured as well (**C**). ^*^P < 0.05 vs. Vector and #P< 0.05 vs. *Gata2*-WT.

To investigate the mechanisms by which GATA2 downregulation induces macrophage pro-inflammatory phenotype and injures multiple processes of macrophage phagocytosis, we conducted bulk RNA-seq of *Gata2*-silenced RAW264.7 cells and found a total of 135 DEGs compared with control cells. Enrichment of the 105 upregulated DEGs in biological processes such as oxidative stress and inflammatory response strongly indicated the pro-inflammatory activation of *Gata2*-silenced RAW264.7 cells. On the other hand, the 30 downregulated DEGs exhibited an involvement in phagocytosis, engulfment, and cholesterol transport (Figure 3A and Supplementary Figure 3A), resembling the result of GO analysis of downregulated DEGs in macrophages from COPD lung tissues (Figure 2A). Therefore, we picked out 6 downregulated DEGs involved in these biological processes, namely *Abca1*, *Abcg1*, *Lpcat3*, *Pacsin1*, *Srebf1*, and *Sting1*, among which the former 5 ones were predicted target genes of GATA2 in GTRD database. Notably, only *Abca1* and *Pacsin1* showed a consistent decline of expression in CSE-treated macrophages and *Gata2*-silenced RAW264.7 cells (Supplementary Figure 3B). ABCA1, a kind of cholesterol transporter capable of regulating cholesterol levels and stability of lipid rafts within the cell membranes, participates in phagocytosis directly together with phagocytic receptor MEGF10 and cytoplasmic adapter protein GULP1 [16, 17]. Besides, ABCA1 has also been documented to amplify the pro-inflammatory signaling through TLR4/MyD88 pathway [18]. In addition, PACSIN1, essential for cytoskeleton rearrangement and autophagosome-lysosome fusion, has been reported to interact with Synaptojanin1 (SYNJ1) to regulate endocytosis and circulation of synaptic vesicle [19]. Thus, we narrowed down the candidate target genes of GATA2 with inflammatory and phagocytic regulatory potential to *Abca1* and *Pacsin1*.

We predicted 3 and 5 GATA2 binding sites in the promoter regions of *Abca1* and *Pacsin1*, respectively in Insect2.0 database [20] and constructed different wild type pGL6-*Abca1*/*Pacsin1*-Luc reporters with these GATA2 binding sites as illustrated in Figure 3B. When co-transfected with *Gata2* overexpression plasmid, only *Abca1*-TFBS2 and *Pacsin1*-TFBS1 reporters showed significantly increased transcriptional activation. Subsequently, we mutated the binding sites in *Abca1*-TFBS2 and *Pacsin1*-TFBS1 reporters and found that overexpression of GATA2 failed to transcriptionally activate the mutant *Abca1*-TFBS2 and *Pacsin1*-TFBS1 reporters (Figure 3C). In addition, *Abca1* and *Pacsin1* were significantly downregulated in *Gata2*-knockdown RAW264.7 cells, owing to the reduction of transcriptional activation of GATA2 to both target genes (Supplementary Figure 3C). Hence, *Abca1* and *Pacsin1* turned out to be the transcriptional target genes of GATA2, both of which have the potential to mediate the effect of GATA2 downregulation on macrophage pro-inflammatory phenotype and phagocytosis.

### ABCA1 mediates the effects of GATA2 downregulation on macrophages by regulating inflammation and ingestion

**Figure 4:**
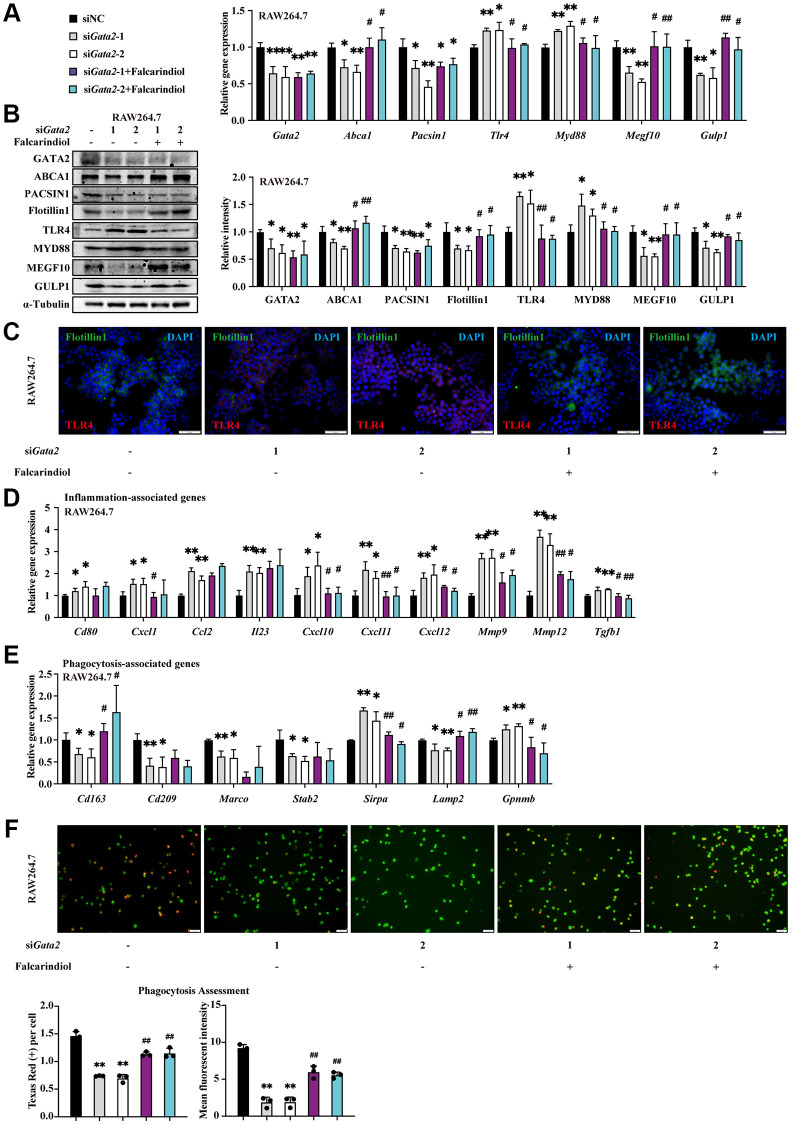
**ABCA1 mediates the effects of GATA2 downregulation on macrophages by regulating inflammation and ingestion.** RAW264.7 cells were incubated with or without si*Gata2* for 48 h, and they were then treated with or without Falcarindiol for another 24 h. mRNA levels of *Gata2, Abca1, Pacsin1, Tlr4, Myd88, Megf10,* and *Gulp1* were assessed by qPCR (**A**) and their protein levels, Flotillin1 was added, were measured by western blotting (**B**) and immunofluorescence staining (**C**) in RAW264.7 cells from each group. mRNA levels of *Cd80, Cxcl1, Ccl2, Il23, Cxcl10, Cxcl11, Cxcl12, Mmp9, Mmp12, Tgfb1, Cd163, Cd209, Marco, Stab2, Sirpa, Lamp2 and Gpnmb* were measured by qPCR (**D**, **E**) in RAW264.7 cells from each group (siNC, black bars; si*Gata2*-1, gray bars; si*Gata2*-2, white bars; si*Gata2*-1+Falcarindiol, purple bars; si*Gata2*-2+Falcarindiol, cyan bars). Phagocytosis assessment was done by Cell Meter^™^ Fluorimetric Phagocytosis Assay Kit (**F**). Scale bars = 50 μm. ^*^P < 0.05 or ^**^P < 0.01 vs. siNC and ^#^*P* < 0.05 or ^##^*P* < 0.01 vs. si*Gata2*.

As a target gene of GATA2, *Abca1* was downregulated in AMs of CS-exposed COPD mouse model (Supplementary Figure 4A). We also observed the activation of the TLR4/MyD88 pathway along with the declines in MEGF10 and GULP1 expression in CSE-treated RAW264.7 cells (Supplementary Figure 4B, 4C). Furthermore, consistent with the decreased number of engulfed beads within CSE-treated RAW264.7 cells in phagocytosis assessment (Figure 2C), the expression levels of Flotillin1, a biomarker of lipid rafts [21], showed a similar CSE-dose-dependent reduction, reflecting instability of macrophage lipid rafts (Supplementary Figure 4C, 4D). Accordingly, we hypothesized that *Abca1* may mediate the effects of GATA2 downregulation on macrophages by regulating inflammation and ingestion through TLR4/MyD88 pathway and MEGF10/GULP1 pathway, respectively. To test this, we employed Falcarindiol, a natural polyacetylene compound able to increase ABCA1 protein expression at the transcriptional and post-transcriptional levels [22], to partly restore the function of ABCA1 as well as the normal activity of its downstream pathways including TLR4/MyD88 and MEGF10/GULP1. As expected, along with the decreased ABCA1 expression, *Gata2* silencing led to the activation of the TLR4/MyD88 pathway as well as the suppression of the MEGF10 and GULP1 expression in RAW264.7 cells, all of which together with the upregulation of pro-inflammatory cytokines exhibited a partial reverse by Falcarindiol (Figure 4A–4D). Meanwhile, in the evaluation of phagocytosis, it was the reduced number of engulfed beads rather than the decreased fluorescence intensity of phagocytized beads in *Gata2*-silenced RAW264.7 cells that was remarkably reversed by administration of Falcarindiol, which was coherent with the partially restored expression of phagocytosis molecules (Figure 4E, 4F). Furthermore, in the context of CSE treatment, Falcarindiol was also able to suppress the expression of pro-inflammatory genes (Supplementary Figure 4E) and sustain phagocytosis in macrophages (Supplementary Figure 4F, 4G). These results suggested that the aberrant expression of inflammation-related molecules and the disruption of ingestion in the early stage of phagocytosis due to CS-induced GATA2 downregulation in macrophages were ascribed to decreased ABCA1 expression-mediated TLR4/MyD88 pathway activation as well as MEGF10/GULP1 pathway suppression, respectively.

### PACSIN1 mediates the effects of GATA2 downregulation on macrophage by regulating phagolysosome formation

**Figure 5:**
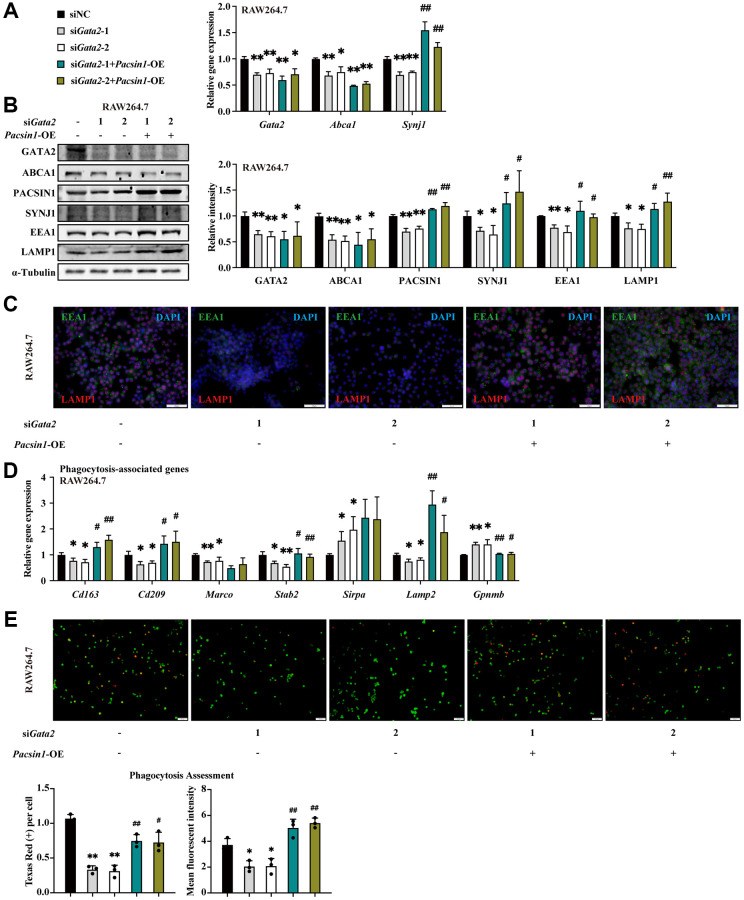
**PACSIN1 mediates the effects of GATA2 downregulation on macrophage by regulating phagolysosome formation.** RAW264.7 cells were co-transfected with or without si*Gata2* and *Pacsin1* overexpression plasmids for 48 h. mRNA levels of *Gata2, Pacsin1* and *Synj1* were assessed by qPCR (**A**) and protein levels of GATA2, ABCA1, PACSIN1, SYNJ1, EEA1, and LAMP1 were assessed by western blotting (**B**) and immunofluorescence staining (**C**) in RAW264.7 cells from each group. mRNA levels of *Cd163, Cd209, Marco, Stab2, Sirpa, Lamp2 and Gpnmb* were measured by qPCR (**D**) in RAW264.7 cells from each group (siNC, black bars; si*Gata2*-1, gray bars; si*Gata2*-2, white bars; si*Gata2*-1+*Pacsin1*-OE, teal bars; si*Gata2*-1+*Pacsin1*-OE, asparagus bars). Phagocytosis assessment was done by Cell Meter^™^ Fluorimetric Phagocytosis Assay Kit (**E**). Scale bars = 50 μm. ^*^P < 0.05 or ^**^P < 0.01 vs. siNC and ^#^*P* < 0.05 or ^##^*P* < 0.01 vs. si*Gata2*.

Similarly, *Pacsin1*, another target gene of GATA2, was significantly decreased in AMs of CS-exposed mice (Supplementary Figure 5A). To verify the potential responsibility of dysregulated PACSIN1 for the impeded formation/maturation of phagolysosome indued by GATA2 downregulation in macrophages, we overexpressed *Pacsin1* in *Gata2*-silenced RAW264.7 cells (Supplementary Figure 5B). Intriguingly, the interactor of PACSIN1, SYNJ1, also showed decline in expression in *Gata2*-sileced RAW264.7 cells, along with the reduction of another two markers of phagosome formation and maturation, EEA1 and LAMP1 [23], which were all restored by *Pacsin1* overexpression (Figure 5A–5C), indicating the improvement of phagolysosome maturation. Coherently, together with the reverse of dysregulated phagocytosis-associated genes (Figure 5D), the reduced fluorescence intensity of phagocytized beads within *Gata2*-silenced RAW264.7 cells was partly reversed by *Pacsin1* overexpression (Figure 5E). Similarly, by upregulating Synj1 (Supplementary Figure 5C–5E), *Pacsin1* overexpression was sufficient to partly resist the damaging effect of CSE on macrophage phagocytosis, especially in the stage of phagolysosome maturation (Supplementary Figure 5F, 5G). Thus, decreased expression of *Pacsin1* due to CS-induced GATA2 downregulation was responsible for the dysfunctional macrophage phagocytosis, especially the late stage of phagocytosis.

### GATA2 improves macrophage inflammatory phenotype and phagocytosis against CSE through transcriptional activation of ABCA1 and PACSIN1

**Figure 6:**
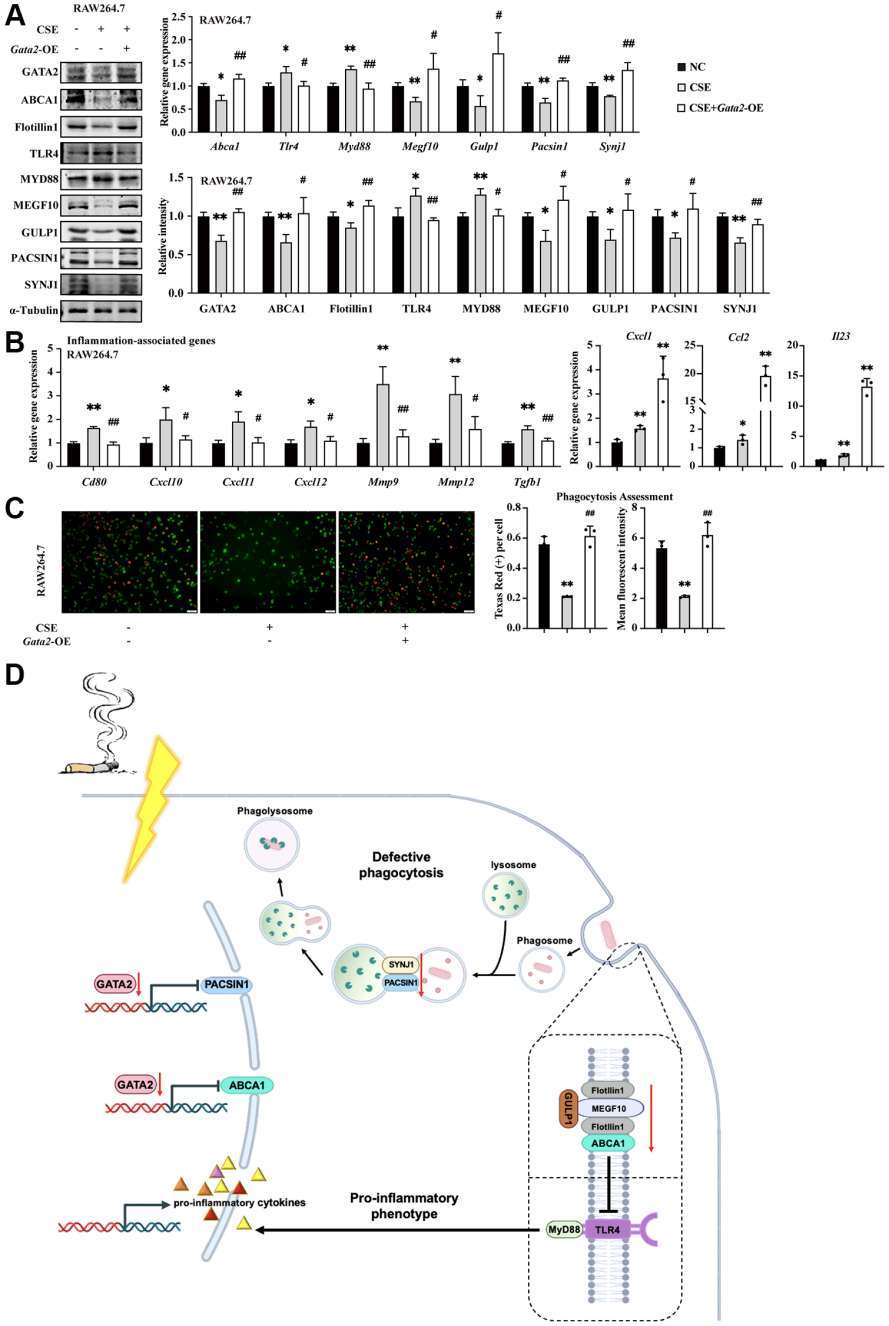
**GATA2 improves macrophage inflammatory phenotype and phagocytosis against CSE through transcriptional activation of ABCA1 and PACSIN1.** RAW264.7 cells were pre-incubated with or without CSE. After 24 h, they were transfected with or without *Gata2* overexpression plasmids for another 48 h. mRNA levels of *Abca1, Tlr4, Myd88, Megf10, Gulp1, Pacsin1,* and *Synj1* were assessed by qPCR and their protein levels, GATA2 and Flotillin1 were added, which were measured by western blotting in RAW264.7 cells from each group (**A**). mRNA levels of *Cd80, Cxcl1, Ccl2, Il23, Cxcl10, Cxcl11, Cxcl12, Mmp9, Mmp12* and *Tgfb1* were assessed by qPCR (**B**) in RAW264.7 cells from each group (NC, black bars; CSE, gray bars; CSE+*Gata2*-OE, white bars). Phagocytosis assessment was done by Cell Meter^™^ Fluorimetric Phagocytosis Assay Kit (**C**). Scale bars = 50 μm. ^*^P < 0.05 or ^**^P < 0.01 vs. NC and ^#^*P* < 0.05 or ^##^*P* < 0.01 vs. CSE. Schematic drawing shows loss of GATA2 in macrophage during COPD is associated with the loss of ABCA1 and PACSIN1. GATA2 directly regulates ABCA1, which is essential for pro-inflammation via TLR4/MYD88 and for phagocytosis via MEGF10 and GULP1. Together with SYNJ1, the decline in PANCIN1 expression, which is another target gene of GATA2, directly hindered the formation and maturation of phagolysosome in macrophages (**D**).

Finally, to further validate the role of GATA2 in modulating macrophage pro-inflammatory phenotype and damaged phagocytosis under the condition of CS exposure, we overexpressed *Gata2* in CSE-treated RAW264.7 cells (Supplementary Figure 6A). As expected, both targets, *Abca1* and *Pacsin1*, were significantly upregulated upon *Gata2* overexpression, together with the repressed TLR4/MyD88 pathway as well as activated MEGF10/GULP1 pathway downstream of ABCA1 and increased expression of PACSIN1 interactor, SYNJ1 (Figure 6A and Supplementary Figure 6B). Correspondingly, the CSE-induced pro-inflammatory phenotype was alleviated as reflected by the decreased expression of inflammation-related molecules (Figure 6B), while the CSE-disrupted phagocytosis was improved as indicated by the increased engulfed beads as well as enhanced fluorescence intensity of phagocytized beads within macrophages in phagocytosis assessment (Figure 6C), together with the restoration of phagocytosis molecules (Supplementary Figure 6C). Similarly, the CSE-induced pro-inflammatory phenotype and phagocytosis dysfunction were mitigated by *Gata2* overexpression in MH-S cells (Supplementary Figure 6D–6G). Collectively, *Gata2* overexpression was sufficient to partially improve the macrophage pro-inflammatory phenotype and phagocytosis against CSE via transcriptionally activating *Abca1* and *Pacsin1*.

## DISCUSSION

In this study, we found that GATA2 was significantly downregulated in pulmonary macrophages from COPD patients and smokers. We clarified that CSE-induced downregulation of GATA2 promoted the pro-inflammatory phenotype and inhibited phagocytosis in macrophages. Mechanistically, due to the downregulation of GATA2, the decreased transcriptional activity of the GATA2 target gene *Abca1* led to a decrease in *Abca1* expression, which facilitated inflammatory secretion through the activation of the TLR4/MYD88 pathway, and directly impaired phagosome formation in macrophages, along with the downregulation of the phagocytic receptor MEGF10 and the cytoplasmic junction protein GULP1. Meanwhile, the decline in the expression of *Pacsin1*, which was confirmed to be another target gene of GATA2, directly hindered the formation and maturation of phagolysosome in macrophages (Figure 6D).

Beyond supporting the generation of diverse myeloid cell progeny [24], GATA2 establishes and maintains a genetic network that even modulate mature immune cells in different pathological contexts. Seemingly contrary to our results, as a signature gene of mast cells (MCs) and basophils, GATA2 was increased in these cells from sputum of COPD patients compared with control individuals, which was also associated with eosinophilic airway inflammation, blood eosinophilia, and decreased lung function [25]. Moreover, Yin et al. [26] found that through a mechanism involving instigating expression of Rab7 and components of both the vacuolar ATPase and NADPH oxidase complexes, upregulation of GATA2 in macrophages from early-stage human atherosclerotic lesions renders the loss of efferocytosis in macrophages, which initiates atherosclerotic plaque formation due to the failed efferocytic clearance of apoptotic cells. As a transcription factor, GATA2 largely depends on the target genes it regulates to execute multiple functions. For example, in fetal liver progenitors, GATA2 restricts action of innate immune mediators via repressing multiple TLR family members including TLR1, TLR2, and TLR6 [27]. Intriguingly, GATA2 was also reported to play a critical role in maintain AM identity and function. Increased expression of GATA2, together with activation of STAT5, AKT, and ERK, were associated with the generation of foamy AMs and the accumulation of pulmonary surfactant resulting from CISH deficiency [28]. In addition, LPS was able to transcriptionally increase IL-1β expression in RAW264.7 cells through upregulating GATA2 [29]. Therefore, the molecular network established by GATA2 based upon different transcriptional targets within pulmonary macrophages could serve as a pleiotropic modulator in response to different stimuli as well as under different conditions, which warrants more extensive studies to fully illustrate its functions and mechanisms.

The pulmonary macrophages from both patients with GATA2 heterozygous mutation (i.e., GATA2 deficiency) and COPD exhibit typical phagocytic dysfunction [1, 5, 8, 10, 30, 31], besides they all show a deficiency/downregulation of GATA2 expression. Furthermore, both clusters of DEGs related to COPD and *Gata2* silencing in pulmonary macrophages were enriched in biological processes associated with inflammation (mTOR, Toll-like receptor, TNF, and Wnt), phagocytosis (endocytosis, lysosome, and phagosome), and lipid metabolism (cholesterol metabolism), reflecting the remarkable resemblance between the functional changes caused by knockdown of *Gata2* and the pathological changes in macrophages during the development of COPD, which suggested that the downregulation of GATA2 is very likely to mediate significant pathological changes in macrophages during COPD development. Meanwhile, the predicted GATA2 target genes from public database also enriched in the aforementioned biological processes, such as positive regulation of inflammatory response, endosome, and lipid metabolic process. Hence, we selected candidate genes related to phagocytosis and inflammation and conducted experimental verification. Such strategy not only enhances the credibility of bioinformatics analysis and avoids potential biases of analysis based on single transcriptome dataset, but also reduces the screening range of candidate target genes, which improves the accuracy and efficiency in exploring functional mechanisms.

As the reverse transporter of cholesterol located on the plasma membrane, Golgi apparatus, and lysosomes of cells [32], ABCA1 is essential for maintaining lipid homeostasis in macrophages. Notably, the *Abca1* knockout mice manifested typical alveolar protein deposition with the existence of foam AMs [18, 33], supporting the role of ABCA1 in the phagocytosis of AMs. Moreover, cholesterol synthesis of AMs has long been reported to be significantly increased in smokers and patients with COPD [34], and their AMs exhibited “foam-like” changes [35], with increased secretion of pro-inflammatory cytokines as well as suppressed phagocytosis. Given that ABCA1, through the transport of PI(4,5)P2, can participate in the regulation of signal transmission along the MyD88 dependent TLR4 pathway [18] and that the localization of TLR4 to cellular membrane relies on cholesterol [35], we proposed that the elevated overall level of cholesterol within macrophages due to the downregulation of ABCA1 can not only amplify the pro-inflammatory signaling through TLR4/MyD88 via accumulated PI(4,5)P2 in lipid rafts, but also increase TLR4 expression and localization in cellular membrane, which may enhance the sensitivity and reactivity of pulmonary macrophages to inhaled pollutants and pathogenic microorganisms, thereby exacerbating the inflammatory response in lung tissue [36]. Additionally, activation of TLR4 was reported to inhibit the transcription factor liver X receptor (LXR) and thus further decrease the expression of ABCA1, creating a vicious circle in macrophages [37]. In addition, as an important component of lipid rafts, ABCA1, along with the phagocytic receptor MEGF10 and the cytoplasmic adapter protein GULP1, also plays a direct role in the formation of phagosomes during the early stage of phagocytosis [16, 17]. Interestingly, our research not only supported the involvement of *Abca1*, as a transcriptional target gene of GATA2, in the macrophage pro-inflammatory phenotype and formation of macrophage phagosomes in the early stage of phagocytosis, but also revealed the consistency of ABCA1, MEGF10, and GULP1 expression in macrophages. Further research is needed to determine whether the downregulation of MEGF10 and GULP1 is regulated directly by GATA2 or indirectly by TLR4/LXR or other pathways activated by *Abca1* downregulation.

Phagocytosis is a complex biological process that involves several sequential steps, including recognition, adhesion, engulfment, components processing, and the formation of phagosomes and phagolysosomes [38]. Beyond the process of engulfment/ingestion, we found that the inhibition of macrophage phagocytosis, by CSE and *Gata2* knockdown, also took place during the formation/maturation of phagolysosome. In our study, *Pacsin1* overexpression significantly reversed the decreased fluorescence intensity of the beads phagocytosed by *Gata2*-silenced RAW264.7 cells, accompanied by a restoration of the expression of EEA1 and LAMP1, suggesting that PACSIN1-SYNJ1, which could facilitate the fusion and transport of vesicles [19, 39], also partially mediated the disruptive effects of GATA2 downregulation on phagocytosis of macrophages. Our findings revealed that GATA2 influences different steps of phagocytosis of pulmonary macrophages during COPD development via different target genes, highlighting the versatile and complex role of GATA2 in COPD pathogenesis, which needs more explorations.

In summary, our study uncovered a decrease in GATA2 expression in pulmonary macrophages of COPD lung tissue, which downregulates its target genes *Abca1* and *Pacsin1*, and therefore facilitates macrophage pro-inflammatory phenotype whereas inhibiting macrophage phagocytosis by affecting the engulfment and the maturation of phagolysosomes. Our findings identified GATA2 as a transcriptional regulatory hub in pulmonary macrophages during COPD development, providing new potential targets for the prevention and treatment of COPD.

## MATERIALS AND METHODS

### Mice and CS exposure

All animal experiments were approved by the Institutional Animal Care and Use Committee at Nanjing Medical University. Male C57BL/6 mice of 8 weeks were purchased from the National Resource Center for Mutant Mice Model Animal Research Center of Nanjing University. Mice were randomly selected and exposed to cigarette smoke (CS) or room air (RA). According to the method of Conlon et al. [40], the control group was placed in a sterile barrier facility and exposed to filtered air. CS exposures were performed via the CSM-100C inhalation exposure apparatus (TOW-int tech, Shanghai, China) using Huangshan Brand cigarettes (China Tobacco Anhui Industrial Co., Ltd., Bengbu, Anhui, China), exposing mice to CS with a total particulate matter concentration (TPM) of 500 mg/m^3^. TPM was real-time monitored by Microdust Pro Real Time Dust Monitor (Casella, Germany) and carbon monoxide (CO) was controlled at 300 parts per million (ppm). Simulating human’s smoking habits, CS exposure was performed for 1 h at a time, twice per day, 7 days a week for 6 months.

### Isolation of mouse alveolar macrophages

According to the method of Zhang et al. [41], after anesthesia, the mouse’s trachea was exposed and incised in the middle. The lung was injected with 0.75 ml of stroke-physiological saline solution via the trachea using a standard 1-ml syringe, after which the bronchoalveolar lavage fluid (BALF) was slowly pumped. This procedure was duplicated for fifteen times. The BALF was centrifuged at 300 g for 5 min. The cell pellet was resuspended in RPMI 1640 medium (Gibco, Waltham, MA, USA) without FBS and incubated for 1 h at 37°C with 5% CO_2_ for preferential adhesion of alveolar macrophages.

### Preparation of aqueous cigarette smoke extract (CSE)

Huangshan Brand cigarettes were used in this study, of which the tar, nicotine, and carbon monoxide contents were 10 mg/cigarette, 0.9 mg/cigarette, and 11 mg/cigarette, respectively. As previously described [42], CSE was prepared by bubbling smoke from 1 cigarette into 10 ml of RPMI 1640 or DMEM (Gibco) at a rate of 1 cigarette per 2 minutes. The pH of CSE was adjusted to 7.2–7.4. After being filtered through a sterile 0.22 μm filter (Merck & Millipore, Darmstadt, Germany), part of CSE preparation was diluted 5–10 times with RPMI 1640 or DMEM and monitored the absorbance at 320 nm (optical density of 0.67 ± 0.01) to choose the best dilution ratio, compared with control medium prepared by bubbling air through 10 ml of culture medium. CSE was diluted with culture medium immediately before use for each experiment.

### Cell culture and treatment

All the cell lines used in this study were purchased from Procell (Wuhan, China). The mouse macrophage cell line RAW264.7 and the mouse alveolar macrophages cell line MH-S were grown in Dulbecco’s Modification of Eagle’s Medium (DMEM) medium (Gibco) containing 10% FBS. The media was supplemented with 100 μg/ml streptomycin, 100 U/ml penicillin, 0.25 μg/ml amphotericin B, and 10 mmol/l HEPES (Sigma-Aldrich, St. Louis, MO, USA). Cells were cultured at 37°C in a humidified 5% CO_2_ atmosphere. For CSE treatment, cells were incubated with or without CSE for 24 h. For Falcarindiol treatment, cells were incubated with or without 24 μM Falcarindiol (MedChemExpress, Shanghai, China) for another 24 h.

### Transient transfection for RAW264.7 and MH-S macrophages

Small interfering RNAs (siRNAs) were designed and synthesized by Ribobio (Guangzhou, Guangdong, China). Overexpression plasmids were designed and synthesized by Genomeditech (Shanghai, China). For transient transfection, Lipofectamine 3000 reagent (Invitrogen, Grand Island, NY, USA) was mixed with siRNAs or plasmids according to the manufacturer’s protocol.

### RNA extraction and RT-PCR assay

Total RNA was extracted from cells and lungs using Trizol reagent (Invitrogen, Carlsbad, CA, USA). RNA was reverse-transcribed into cDNA with HiScript II Q RT SuperMix for qPCR (Vazyme, Nanjing, China). Quantification RT-PCR was performed using ChamQTM Universal SYBR qPCR Master Mix (Vazyme) and Step One PlusTM Real-Time PCR System (Applied Biosystems, Foster City, CA, USA). Arpppo was used as internal standards for mRNAs. The primers used are displayed in Supplementary Table 1.

### Western blot analysis

Western blotting was performed as previously described [43]. Individual immunoblots were probed with rabbit anti-GATA2 mAb (Abcam, New Territories, Hong Kong, China) diluted 1:1000, rabbit anti-ABCA1 mAb (Cell Signaling Technology, Danvers, MA, USA) diluted 1:1000, rabbit anti-Flotillin1 mAb (Abcam, New Territories, Hong Kong, China) diluted 1:5000, rabbit anti-TLR4 pAb (Proteintech, Wuhan, China) diluted 1:1000, mouse anti-MYD88 mAb (Proteintech) diluted 1:5000, rabbit anti-MEGF10 pAb (Merck & Millipore) diluted 1:800, rabbit anti-GULP1 pAb (Proteintech) diluted 1:2000, rabbit anti-PACSIN1 pAb (Proteintech) diluted 1:1000, rabbit anti-Synaptojanin-1 Ab (Cell Signaling Technology, USA) diluted 1:1000, mouse anti-EEA1 mAb (Proteintech) diluted 1:10000, rabbit anti-Lamp1 pAb (ABclonal, Woburn, MA, USA) diluted 1:1000, mouse anti-α-Tubulin mAb (Sigma Aldrich) diluted 1:4000 in 2.5% (wt/vol.) non-fat dried milk in Tris-buffered saline with Tween-20 (TBST) buffer.

### Immunofluorescence staining

Immunofluorescence staining was performed as described previously [43]. In brief, it was performed using primary antibodies to GATA2 (1:100, Proteintech), F4/80 (1:50, Abcam), Flotillin1 (1:500, Abcam), TLR4 (1:100, Abcam), EEA1 (1:4000, Proteintech), LAMP1 (1:100, ABclonal) and associated fluorescein (FITC)- and Cy3-conjugated secondary antibodies (Jackson ImmunoResearch, West Grove, PA, USA) per manufacturer instructions. Stained sections were imaged using OLYMPUS automated fluorescence microscope BX63 (Olympus, Shinjuku, Tokyo, Japan).

### Assessment of phagocytosis for RAW264.7 and MH-S macrophages

The phagocytosis of macrophages was measured using the Cell Meter Fluorimetric Phagocytosis Assay Kit (AAT Bioquest, Sunnyvale, CA, USA) according to the manufacturer’s instructions. To analyze the phagocytic activity, Texas Red signal indicates the endocytosis of Protonex 600 Red-Latex bead conjugate in macrophages, while CytoTrace Green is applied to evaluate the cell numbers as an internal control. The images were taken using OLYMPUS automated fluorescence microscope BX63 (Olympus, Shinjuku, Tokyo, Japan).

### Transcriptome sequencing

Total RNA was isolated and purified using TRIzol reagent (Invitrogen, Carlsbad, CA, USA) following the manufacturer’s procedure. The RNA amount and purity of each sample was quantified using NanoDrop ND-1000 (NanoDrop, Wilmington, DE, USA). The RNA integrity was assessed by Bioanalyzer 2100 (Agilent, Santa Clara, CA, USA) with RIN number >7.0, and confirmed by electrophoresis with denaturing agarose gel. At last, we performed the 2 × 150 bp paired-end sequencing (PE150) on an Illumina Novaseq^™^ 6000 (LC-Bio Technology Co., Ltd., Hangzhou, China) following the vendor’s recommended protocol. The differentially expressed mRNAs were selected with fold change >2 or fold change <0.5 and with parametric F-test comparing nested linear models (*p*-value < 0.05) by R package edgeR (https://bioconductor.org/packages/release/bioc/html/edgeR.html).

### Luciferase reporter assay

Mouse aortic endothelial cells (MAECs) were seeded in 6-well plates at a confluence of 70%. 2.8 μg of the Gata2 over-expression plasmid, firefly and renilla luciferase reporter plasmid (vector: pGL-6) were added for co-transfection. After 48 h, luciferase activities were detected with the Dual-Luciferase Reporter Assay System (Beyotime, Haimen, China). Relative activity was assessed using the ratio of the firefly luciferase signal to the renilla luciferase signal.

### Statistical analysis

At least three independent experiments were performed. Comparisons were performed using the Student’s *t*-test between two groups or ANOVA in multiple groups. Results were presented as means ± SEM. A value of *P* < 0.05 was considered statistically significant.

## Supplementary Materials

Supplementary Figures

Supplementary Table 1
