# Supplementary material for: GATA2 downregulation contributes to pro-inflammatory phenotype and defective phagocytosis of pulmonary macrophages in chronic obstructive pulmonary disease
Source: Aging (Albany NY). 2024 Oct 7;16(19):12928–51. doi: 10.18632/aging.206129 (PMC11501382; doi:10.18632/aging.206129)
Supplement: Supplementary Figures [file aging-16-206129-s001.pdf]

SUPPLEMENTARY FIGURES

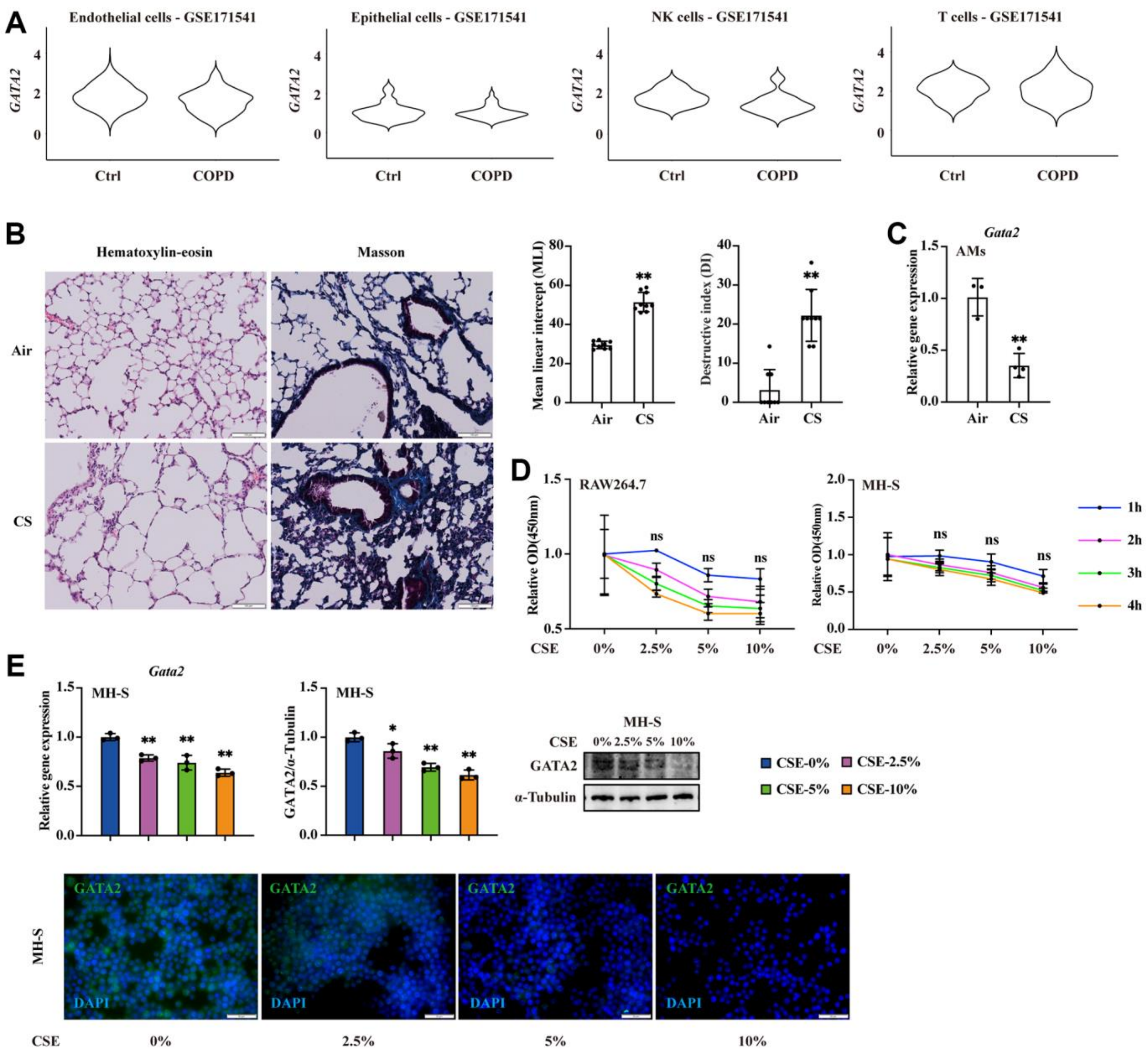

Supplementary Figure 1. GATA2 is downregulated in pulmonary macrophages during the development of CS-triggered COPD. mRNA levels of *GATA2* were analyzed in database GSE171541 (**A**). Eight-week-old mice were exposed to cigarette smoke (CS) or room air. The lungs were harvested and subjected to H&E staining, Masson trichrome staining and measurements of mean linear intercept (MLI) and destruction index (DI) (**B**). Scale bars = 100 µm. mRNA levels of *Gata2* were measured by qPCR (**C**) in alveolar macrophages from BALF of mice. Cell activity was determined by CCK-8 method (**D**) in RAW264.7 and MH-S cells treated with CSE for 24 h from each group (1 hour, blue lines; 2 hour, magenta lines; 3 hour, green lines; 4 hour, orange lines). mRNA and protein levels of GATA2 were measured by qPCR, western blotting, and immunofluorescence staining (**E**) in MH-S cells treated with CSE for 24 h from each group (CSE-0%, blue bars; CSE-2.5%, magenta bars; CSE-5%, green bars; CSE-10%, orange bars). $^{*}P < 0.05$ or $^{**}P < 0.01$ vs. Air 5–10 animals per group or CSE-0%.

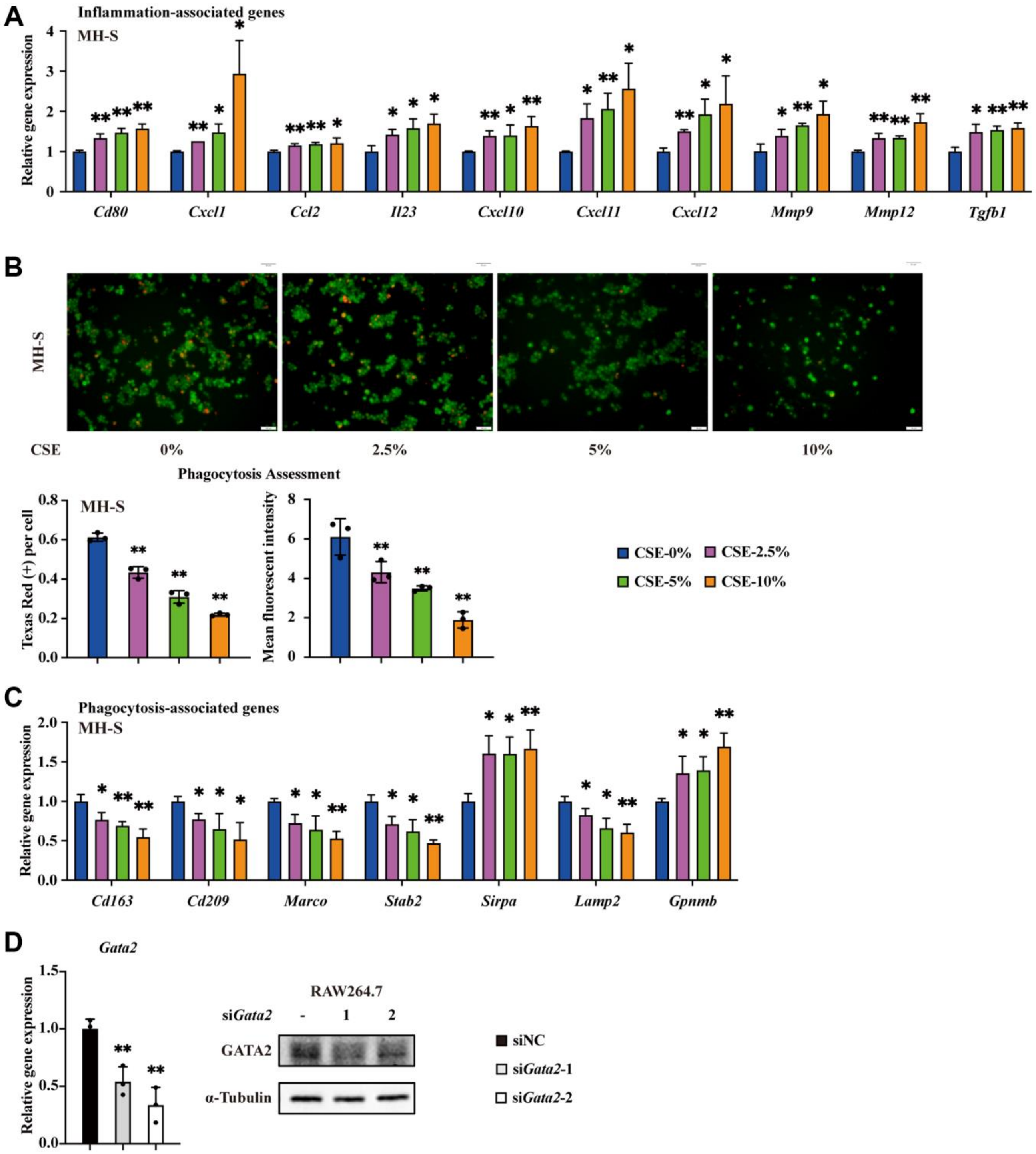

Supplementary Figure 2. Downregulation of GATA2 leads to pro-inflammatory phenotype and impaired phagocytosis in macrophage. mRNA levels of *Cd80, Cxcl1, Ccl2, Il23, Cxcl10, Cxcl11, Cxcl12, Mmp9, Mmp12,* and *Tgfb1* were measured by qPCR (**A**) in CSE-treated MH-S cells from each group. Phagocytosis assessment in MH-S cells was done by Cell Meter™ Fluorimetric Phagocytosis Assay Kit. The images were taken using fluorescence microscopy and the average number of engulfed beads within every cell and the mean fluorescent intensity of Texas Red were calculated (**B**). Scale bars = 50 μm. mRNA levels of *Cd163, Cd209, Marco, Stab2, Sirpa, Lamp2,* and *Gpnmb* were measured by qPCR (**C**) in CSE-treated MH-S cells from each group (CSE-0%, blue bars; CSE-2.5%, magenta bars; CSE-5%, green bars; CSE-10%, orange bars). $^{*}P$ < 0.05 or $^{**}P$ < 0.01 vs. CSE-0%. The efficiency of siRNA was assessed by qPCR and western blotting (**D**) (siNC, black bars; si*Gata2*-1, gray bars; si*Gata2*-2, white bars). $^{*}P$ < 0.05 or $^{**}P$ < 0.01 vs. siNC.

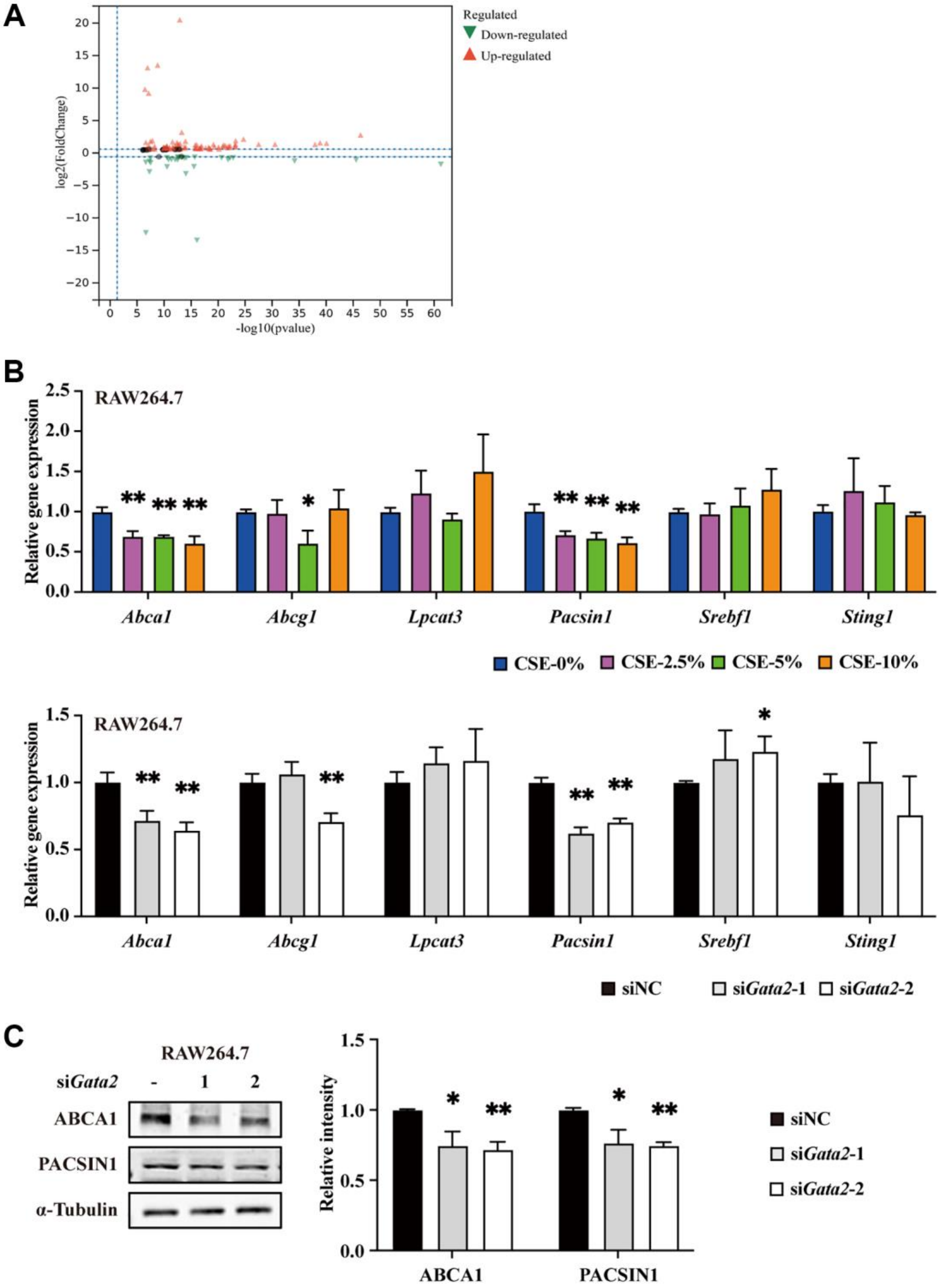

Supplementary Figure 3. GATA2 directly promotes the transcriptional activity of *Abca1* and *Pacsin1*. Volcano plots for DEGs expressed in RAW264.7 cells with or without *Gata2* knockdown. Red dots represent upregulated genes and green dots represent downregulated genes (**A**). mRNA levels of *Abca1, Abcg1, Lpcat3, Pacsin1, Srebf1,* and *Sting1* were measured by qPCR (**B**) in RAW264.7 cells from each group (CSE-0%, blue bars; CSE-2.5%, magenta bars; CSE-5%, green bars; CSE-10%, orange bars; siNC, black bars; si*Gata2*-1, gray bars; si*Gata2*-2, white bars). Protein levels of ABCA1 and PACSIN1 were assessed by western blotting (**C**) in RAW264.7 cells from each group (siNC, black bars; si*Gata2*-1, gray bars; si*Gata2*-2, white bars). $^{*}P < 0.05$ or $^{**}P < 0.01$ vs. siNC.

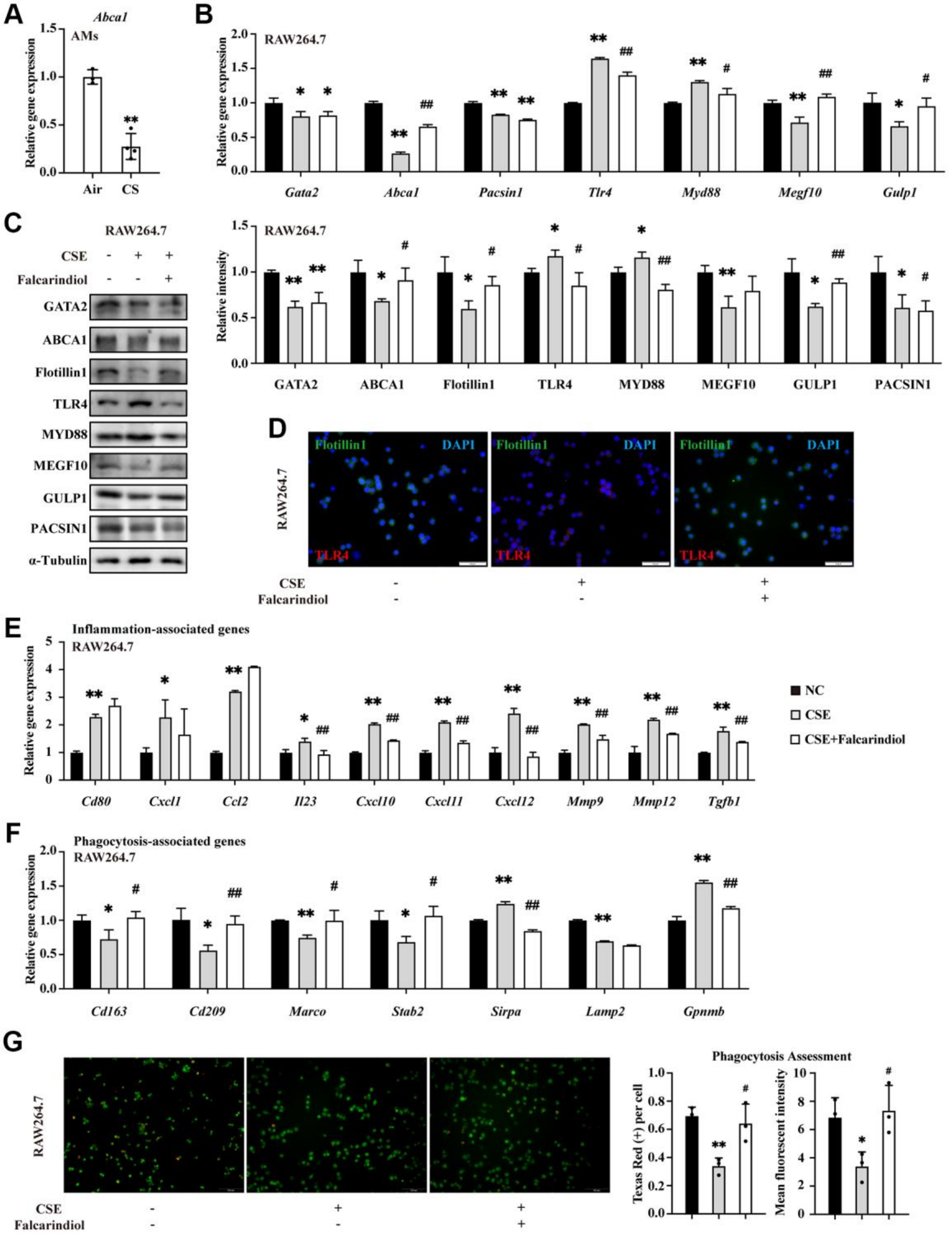

Supplementary Figure 4. ABCA1 mediates the effects of GATA2 downregulation on macrophages by regulating inflammation and ingestion. mRNA levels of *Abca1* were measured by qPCR in alveolar macrophages from BALF of mice (**A**). $^{*}P < 0.05$ or $^{**}P < 0.01$ vs. Air 5–10 animals per group. RAW264.7 cells were incubated with or without CSE for 24 h, and they were then treated with or without Falcarindiol for another 24 h. mRNA levels of *Gata2, Abca1, Pacsin1, Tlr4, Myd88, Megf10,* and *Gulp1* were assessed by qPCR (**B**) and their protein levels, Flotillin1 was added, which were measured by western blotting (**C**) and immunofluorescence staining (**D**) in RAW264.7 cells from each group. mRNA levels of *Cd80, Cxcl1, Ccl2, Il23, Cxcl10, Cxcl11, Cxcl12, Mmp9, Mmp12, Tgfb1, Cd163, Cd209, Marco, Stab2, Sirpa, Lamp2* and *Gpnmb* were measured by qPCR (**E**, **F**) in RAW264.7 cells from each group (NC, black bars; CSE, gray bars; CSE+Falcarindiol, white bars). Phagocytosis assessment was done by Cell Meter™ Fluorimetric Phagocytosis Assay Kit (**G**). Scale bars = 50 μm. $^{*}P < 0.05$ or $^{**}P < 0.01$ vs. NC and $^{\#}P < 0.05$ or $^{\#\#}P < 0.01$ vs. CSE.

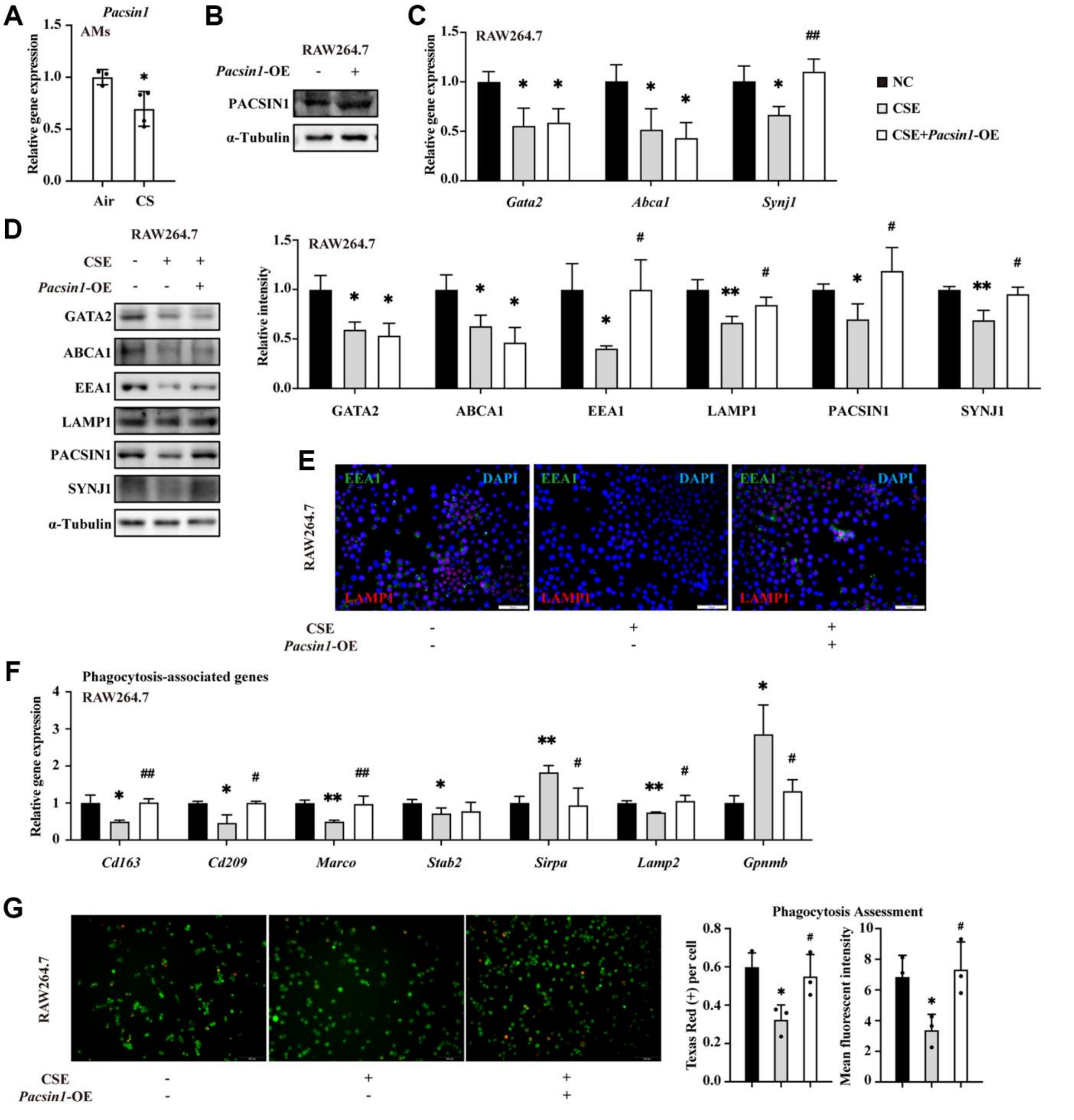

Supplementary Figure 5. PACSIN1 mediates the effects of GATA2 downregulation on macrophage by regulating phagolysosome formation. mRNA levels of *Pacsin1* were measured by qPCR in alveolar macrophages from BALF of mice (**A**). $^{*}P < 0.05$ or $^{**}P < 0.01$ vs. Air 5–10 animals per group or CSE-0%. The efficiency of *Pacsin1* overexpression plasmids was assessed by western blotting (**B**). RAW264.7 cells were incubated with or without CSE for 24 h, and then transfected with or without *Pacsin1* overexpression plasmids for 48 h. mRNA levels of *Gata2, Pacsin1* and *Synj1* were assessed by qPCR (**C**) and protein levels of GATA2, ABCA1, PACSIN1, SYNJ1, EEA1, and LAMP1 were assessed by western blotting (**D**) and immunofluorescence staining (**E**) in RAW264.7 cells from each group. mRNA levels of *Cd163, Cd209, Marco, Stab2, Sirpa, Lamp2 and Gpnmb* were measured by qPCR (**F**) in RAW264.7 cells from each group (NC, black bars; CSE, gray bars; CSE+*Pacsin1*-OE, white bars). Phagocytosis assessment was done by Cell Meter™ Fluorimetric Phagocytosis Assay Kit (**G**). Scale bars = 50 µm. $^{*}P < 0.05$ or $^{**}P < 0.01$ vs. NC and $^{\#}P < 0.05$ or $^{\#\#}P < 0.01$ vs. CSE.

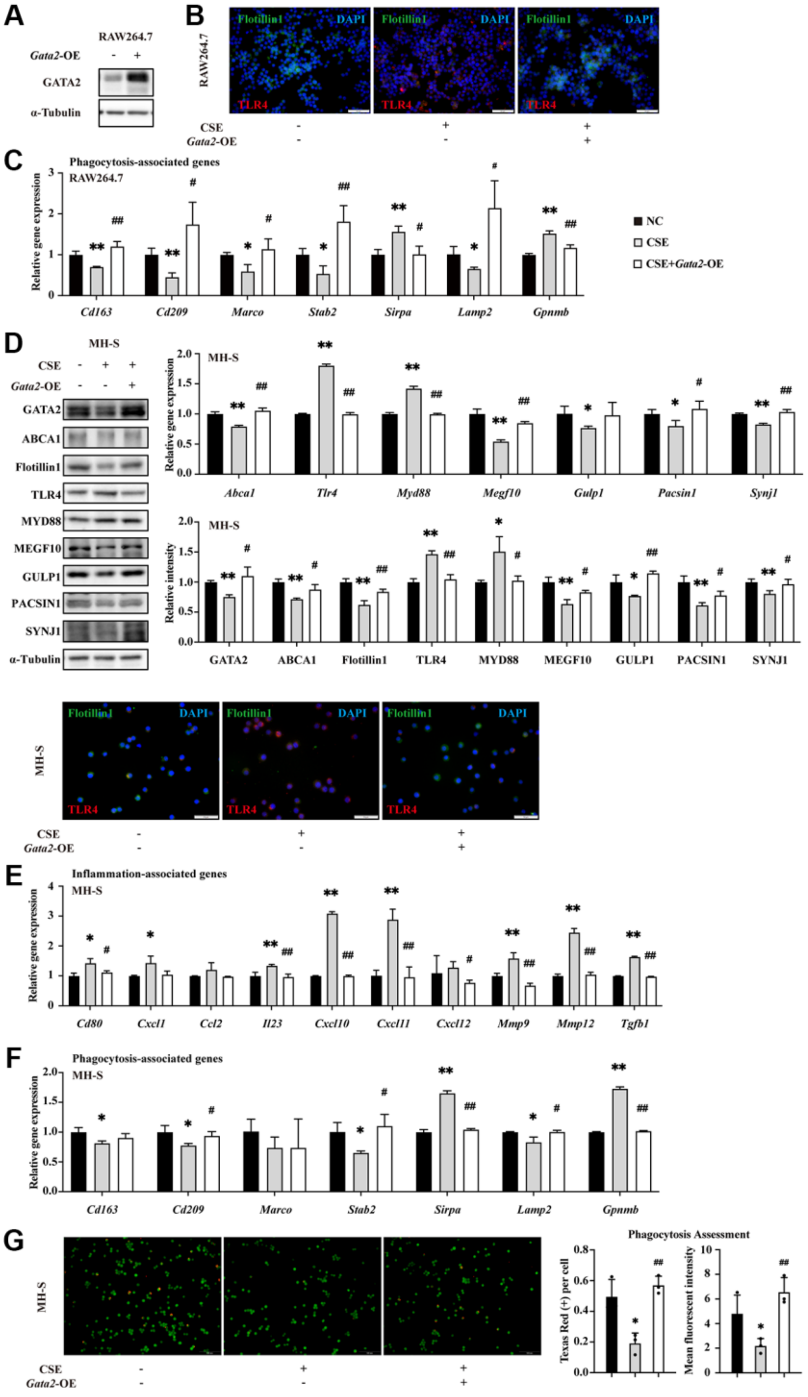

Supplementary Figure 6. GATA2 improves macrophage inflammatory phenotype and phagocytosis against CSE through transcriptional activation of ABCA1 and PACSIN1. The efficiency of *Gata2* overexpression plasmids was assessed by western blotting (**A**). Protein levels of Flotllin1 and TLR4 were measured by immunofluorescence staining (**B**). Scale bars = 50 μm. mRNA levels of *Cd163, Cd209, Marco, Stab2, Sirpa, Lamp2,* and *Gpnmb* were assessed by qPCR (**C**) in RAW264.7 cells from each group (NC, black bars; CSE, gray bars; CSE+*Gata2*-OE, white bars). $^{*}P < 0.05$ or $^{**}P < 0.01$ vs. NC and $^{\#}P < 0.05$ or $^{\#\#}P < 0.01$ vs. CSE. MH-S cells were pre-incubated with or without CSE. After 24 h, they were transfected with or without *Gata2* overexpression plasmids for another 48 h. mRNA levels of *Abca1, Tlr4, Myd88, Megf10, Gulp1, Pacsin1,* and *Synj1* were assessed by qPCR and their protein levels, GATA2 and Flotillin1 were added, which were measured by western blotting and immunofluorescence staining in MH-S cells from each group (**D**). mRNA levels of *Cd80, Cxcl1, Ccl2, Il23, Cxcl10, Cxcl11, Cxcl12, Mmp9, Mmp12, Tgfb1, Cd163, Cd209, Marco, Stab2, Sirpa, Lamp2* and *Gpnmb* were assessed by qPCR (**E**, **F**) in MH-S cells from each group (NC, black bars; CSE, gray bars; CSE+*Gata2*-OE, white bars). Phagocytosis assessment was done by Cell Meter™ Fluorimetric Phagocytosis Assay Kit (**G**). Scale bars = 50 μm. $^{*}P < 0.05$ or $^{**}P < 0.01$ vs. NC and $^{\#}P < 0.05$ or $^{\#\#}P < 0.01$ vs. CSE.
