# Supplementary material for: GATA2 downregulation contributes to pro-inflammatory phenotype and defective phagocytosis of pulmonary macrophages in chronic obstructive pulmonary disease
Source: Aging (Albany NY). 2024 Oct 7;16(19):12928–51. doi: 10.18632/aging.206129 (PMC11501382; doi:10.18632/aging.206129)
Supplement: Supplementary Table 1 [file aging-16-206129-s002.pdf]

SUPPLEMENTARY TABLE

Supplementary Table 1. Details of primers used.

Gene		Primer (5′–3′)
Mouse *Gata2*	Forward	CACCCCGCCGTATTGAATG
	Reverse	CCTGCGAGTCGAGATGGTTG
Mouse *Cxcl1*	Forward	CTGGGATTCACCTCAAGAACATC
	Reverse	CAGGGTCAAGGCAAGCCTC
Mouse *Ccl2*	Forward	TTAAAAACCTGGATCGGAACCAA
	Reverse	GCATTAGCTTCAGATTTACGGGT
Mouse *Il23*	Forward	ATGCTGGATTGCAGAGCAGTA
	Reverse	ACGGGGCACATTATTTTTAGTCT
Mouse *Cxcl10*	Forward	CCAAGTGCTGCCGTCATTTTC
	Reverse	GGCTCGCAGGGATGATTTCAA
Mouse *Cxcl11*	Forward	GGCTTCCTTATGTTCAAACAGGG
	Reverse	GCCGTTACTCGGGTAAATTACA
Mouse *Cxcl12*	Forward	TGCATCAGTGACGGTAAACCA
	Reverse	CACAGTTTGGAGTGTTGAGGAT
Mouse *Mmp9*	Forward	CTGGACAGCCAGACACTAAAG
	Reverse	CTCGCGGCAAGTCTTCAGAG
Mouse *Mmp12*	Forward	CTGCTCCCATGAATGACAGTG
	Reverse	AGTTGCTTCTAGCCCAAAGAAC
Mouse Tgfb1	Forward	CTCCCGTGGCTTCTAGTGC
	Reverse	GCCTTAGTTTGGACAGGATCTG
Mouse *Cd163*	Forward	TCTCAGTGCCTCTGCTGTCA
	Reverse	TCTTCCTTGACTCTGACCGC
Mouse *Cd209*	Forward	CTGACAGATGAGCTTACGTCCA
	Reverse	CACAGGCGGAAGAGTTCAGTC
Mouse *Marco*	Forward	AGAGGGAGAGCACTTAGCAG
	Reverse	CTGTGCCCCGACAATTCACAT
Mouse *Stab2*	Forward	ATTGCTCTGGCTGCCTACTC
	Reverse	GTTGGCTGGCTTCTCACATC
Mouse *Sirpa*	Forward	TCGAGTGATCAAGGGAGCAT
	Reverse	CCTGGACACTAGCATACTCTGAG
Mouse *Lamp2*	Forward	TGTATTTGGCTAATGGCTCAGC
	Reverse	TATGGGCACAAGGAAGTTGTC
Mouse *Gpnmb*	Forward	TGCCAAGCGATTTCGTGATGT
	Reverse	GCCACGTAATTGGTTGTGCTC
Mouse *Abca1*	Forward	GCTTGTTGGCCTCAGTTAAGG
	Reverse	GTAGCTCAGGCGTACAGAGAT
Mouse *Abcg1*	Forward	CTTTCCTACTCTGTACCCGAGG
	Reverse	CGGGGCATTCCATTGATAAGG

Mouse *Lpcat3*	Forward	GACGGGGACATGGGAGAGA
	Reverse	GTAAAACAGAGCCAACGGGTAG
Mouse *Pacsin1*	Forward	GAGGTGGGGAACTACAAGCG
	Reverse	GTATGCCTTCTCGATCTTGGC
Mouse *Srebf1*	Forward	TGACCCGGCTATTCCGTGA
	Reverse	CTGGGCTGAGCAATACAGTTC
Mouse *Sting1*	Forward	GGTCACCGCTCCAAATATGTAG
	Reverse	CAGTAGTCCAAGTTCGTGCGA
Mouse *Tlr4*	Forward	ATGGCATGGCTTACACCACC
	Reverse	GAGGCCAATTTTGTCTCCACA
Mouse *Myd88*	Forward	AGGACAAACGCCGGAACTTTT
	Reverse	GCCGATAGTCTGTCTGTTCTAGT
Mouse *Megf10*	Forward	GAAGACCCCAACGTATGCAG
	Reverse	CGGTGCAGCTTGTGTAGTAGA
Mouse *Gulp1*	Forward	ACAGAAGTTGTGAGAGATGCTG
	Reverse	GCAGTTGTGTTGAACCTCCTTT
Mouse *Synj1*	Forward	GTGTTCGTATGTCAAGGGGATT
	Reverse	CCTTCCTTAGACCCAAGCAGAT
Arpppo	Forward	GAAACTGCTGCCTCACATCCG
	Reverse	GCTGGCACAGTGACCTCACACG
